# A Low Forced Vital Capacity (FVC)/Diffusing Capacity of the Lung for Carbon Monoxide (DLCO) Ratio Increases Clinical Suspicion for Fibrotic Hypersensitivity Pneumonitis (FHP) Over Idiopathic Pulmonary Fibrosis (IPF)

**DOI:** 10.7759/cureus.73008

**Published:** 2024-11-04

**Authors:** Tannam S Vongvivitpatana, Anoop M Nambiar

**Affiliations:** 1 Internal Medicine, University of Colorado, Denver, USA; 2 Internal Medicine, University of Texas Health Science Center at San Antonio, San Antonio, USA; 3 Medicine/Pulmonary Disease, University of Texas Health Science Center at San Antonio, San Antonio, USA; 4 Internal Medicine/Pulmonary Disease, Audie L. Murphy VA Hospital, South Texas Veterans Health Care System, San Antonio, USA

**Keywords:** diffusion capacity of the lung, fibrotic hypersensitivity pneumonitis, forced vital capacity, idiopathic pulmonary fibrosis, interstitial lung disease, pulmonary function test

## Abstract

Background and objective

Fibrotic Hypersensitivity Pneumonitis (FHP) and idiopathic pulmonary fibrosis (IPF) are interstitial lung diseases (ILDs) that are challenging to differentiate with prognostic and therapeutic implications. Clinical observations suggest that patients with FHP may have a lower baseline ratio of forced vital capacity (FVC) to the diffusing capacity of the lung for carbon monoxide (DLCO), or FVC/DLCO (F/D) ratio, than patients with IPF. In light of this, we aimed to determine whether patients with FHP have a significantly lower baseline F/D ratio than patients with IPF.

Methods

A retrospective chart review was performed at a single academic ILD center. Patients with a probable or definite diagnosis of FHP or IPF were considered for inclusion, while patients with poor-quality pulmonary function tests (PFTs) were excluded. The data collected included demographics, diagnosis modality, FVC and DLCO values within six months of diagnosis, as well as hemoglobin levels within three months of PFTs. Baseline F/D ratios were calculated using each patient’s FVC percentage of predicted value divided by the DLCO percentage of predicted value adjusted for hemoglobin when available. One-tailed independent two-sample T-tests were performed.

Results

Eighty-nine patients met the inclusion criteria: 39 (44%) with FHP and 50 (56%) with IPF. The mean baseline F/D ratio was significantly lower for patients with FHP (M = 1.24, 95% CI: 1.14, 1.33) than for patients with IPF (M = 1.44, 95% CI: 1.31, 1.57, T(87) = 2.23, p = 0.014). A secondary analysis excluding patients with pulmonary hypertension and resting hypoxemia was performed, yielding 72 patients: 32 (44%) with FHP and 40 (56%) with IPF. The mean baseline F/D ratio was significantly lower for patients with FHP (M = 1.22, 95% CI: 1.12, 1.31) compared to patients with IPF (M = 1.37, 95% CI: 1.27, 1.46, T (70) = 2.37, p = 0.01).

Conclusions

In patients with probable to definite FHP versus IPF, the baseline F/D ratio was significantly lower in patients with FHP, even after excluding patients with coexisting pulmonary hypertension and resting hypoxemia. A lower baseline F/D ratio may be a novel, clinic-ready index to heighten clinical suspicion for FHP compared to IPF. Further larger prospective studies are needed to validate our findings.

## Introduction

Fibrotic hypersensitivity pneumonitis (FHP) and idiopathic pulmonary fibrosis (IPF) are two common yet distinct causes of interstitial lung disease (ILD); these conditions are challenging to differentiate diagnostically as they can share similar clinical, radiographic, pathological, and physiological characteristics. The diagnostic workup for both diseases is typically lengthy and invasive, requiring a thorough review of patient history, serological testing, high-resolution CT (HRCT) scans, pulmonary function tests (PFTs), lung biopsies, and multidisciplinary discussions [1-3]. Since the timely diagnosis of FHP versus IPF has significant prognostic and therapeutic implications, there is a need for a new, readily available, and non-invasive tool that can help guide clinical suspicion for FHP over IPF.

Previous studies have attempted to distinguish IPF, nonspecific interstitial pneumonia, and FHP with HRCT alone, but were only successful in confidently distinguishing between these diseases in 50% of patients [4]. Another study evaluated the use of HRCT findings to differentiate between FHP and IPF, noting that the “headcheese sign” (also called the “three-density” pattern) is more specific for FHP than mosaic attenuation, which are both common findings associated with FHP [5]. However, no studies evaluating a tool outside of the existing workup for differentiating FHP from IPF are available in the literature.

Anecdotal observations in the clinic suggest that patients with FHP may have a lower baseline ratio of forced vital capacity (FVC) to diffusing capacity of the lung for carbon monoxide (DLCO), or FVC/DLCO (F/D) ratio, than patients with IPF. It remains unknown whether patients with FHP have a lower baseline F/D ratio than patients with IPF. Hence, the purpose of this study is to elucidate whether patients with FHP have a significantly lower baseline F/D ratio than patients with IPF.

This article was previously presented as an oral presentation at the CHEST 2023 conference in Honolulu, HI on October 8, 2023.

## Materials and methods

This study involved a retrospective chart review performed at a single academic ILD center. Inclusion criteria included patients with a probable or definite diagnosis of FHP or IPF as per the American Thoracic Society (ATS) consensus guidelines [1,2]. Exclusion criteria included patients with poor-quality PFTs, as indicated by grades C or lower per ATS and ATS/European Respiratory Society guidelines for FVC and DLCO measurement, respectively, patients without FVC and DLCO values within six months of diagnosis, and/or patients with a diagnosis other than FHP or IPF [6,7]. IPF is typically a diagnosis of exclusion and is defined as a progressive, fibrosing, and chronic interstitial pneumonia of unknown etiology with a typical usual interstitial pneumonia pattern [2]. FHP is normally defined as a dysregulated immune or inflammatory and fibrotic disease related to environmental antigens [1,3].

Key prospectively collected data included demographics, comprising age, sex, race, ethnicity, and smoking status, diagnosis modalities such as HRCT, histopathology, and/or multidisciplinary discussion, FVC and DLCO values within six months of confirmed diagnosis, hemoglobin within three months of PFTs, and presence of supplemental oxygen use at the time of diagnosis and PFTs. Hemoglobin values within three months of PFTs were collected to adjust DLCO values for hemoglobin. The cut-off of six months from a confirmed diagnosis for FVC and DLCO values was utilized as it is expected to not change for PFTs drastically during this timeframe, while a period over six months could allow for substantial change of values due to varying efficacy of treatments, worsening or improvement of disease, or other comorbidities. HRCT, histopathology, and multidisciplinary discussion results were referenced with the ATS guidelines for diagnosis of FHP and IPF to ensure diagnoses in patients’ medical records were consistent with the most recent guidelines and recommendations [1,2].

Evidence suggestive of pulmonary hypertension on echocardiogram (defined by tricuspid regurgitant jet velocity ≥2.8 m/s, estimated pulmonary artery systolic pressure over 35 mmHg, and right ventricle wall abnormalities) and/or confirmed by right heart catheterization (mean pulmonary artery pressure ≥ 20 mmHg, PVR > 2 Wood units) was also recorded [8,9]. A secondary analysis was performed excluding patients with pulmonary hypertension as defined above and/or evidence of resting hypoxemia defined by the use of supplemental oxygen at rest at the time of PFTs as these two conditions are potential confounders. Pulmonary hypertension is expected to decrease DLCO values due to the altered hemodynamics including decreased capillary blood flow and volume as well as structural vascular changes associated with the disease, such as fibroproliferation that thickens the capillaries and affects membrane diffusion [10]. The use of supplemental oxygen at rest may increase DLCO values due to increased oxygen concentrations.

Baseline F/D ratios were initially calculated using each patient’s FVC percentage of predicted value divided by the DLCO percent of predicted value adjusted for hemoglobin when available (F/D (%pred) = FVC%pred/DLCO%pred). The percentage of predicted values for FVC and DLCO were first utilized in calculating the F/D ratio as these values are typically larger and simpler to work with than the absolute values, allowing for practical calculations and estimates in the clinical setting. The DLCO percentage of predicted value adjusted for hemoglobin was calculated using the following standard formulas: Adjusted DLCO (females) = Predicted DLCO x ((1.7 x Hgb)/(9.38 + Hgb)), Adjusted DLCO (for males ≥15 years) = Predicted DLCO x ((1.7 x Hgb)/(10.22 + Hgb)) [11]. One-tailed independent two-sample T-tests were performed with a p-value less than 0.05.

In light of the evidence against the use of race and ethnicity in PFTs and the official ATS statement, F/D ratios were also calculated using each patient’s FVC absolute value divided by the DLCO absolute value (F/D (absolute) = FVCabs/DLCOabs) [12]. Race and ethnicity were collected to establish the study population’s characteristics and to establish if race-specific equations for FVC and DLCO would influence the results found. This study was reviewed by the Institutional Review Board at the University of Texas Health Sciences Center San Antonio and was determined to be exempt on 11/01/2021 under the following directive in DHHS Regulation 45 CFR, Category 4(iii): secondary research uses of identifiable private information.

## Results

Baseline characteristics per the primary analysis of FHP and IPF cohorts are shown in Table 1. In this set of patients, males and females were almost equally represented in the FHP cohort (51% female) while males were predominant in the IPF cohort (76%), consistent with the general population trends. Furthermore, Table 2 demonstrates the similarity of disease severity in the FHP and IPF cohorts as there were similar proportions of patients in each GAP-IPF (GAP = gender, age, and physiology) scoring stage, which are based on gender, age, and FVC and DLCO percentage predicted values to estimate one-year mortality [13]. Table 3 shows ILD-GAP scores, which utilize similar criteria as GAP-IPF but with adjustments in the score for observed differences in mortality in other ILDs compared to IPF, with a score adjustment of -2 for FHP [14].

**Table 1:** Characteristics of FHP and IPF cohorts GAP stages utilized GAP-IPF for IPF and GAP-ILD for FHP DLCO: diffusion capacity of lungs for carbon monoxide; FHP: fibrotic hypersensitivity pneumonitis; FVC: forced vital capacity; GAP: gender, age, and physiology; HRCT: high-resolution computed tomography; ILD: interstitial lung disease; IPF: idiopathic pulmonary fibrosis

	Fibrotic hypersensitivity pneumonitis (FHP)	Idiopathic pulmonary fibrosis (IPF)
Number of patients (%)	39 (44%)	50 (56%)
Age, years, mean	68	72
Males, n (%)	20 (51%)	38 (76%)
Non-Hispanic/Hispanic, n (%)	19 (49%), 10 (26%)	31 (62%), 12 (24%)
Tobacco use, n (%)	14 (36%)	25 (50%)
Supplemental oxygen at rest, n (%)	6 (15%)	4 (8%)
FVC absolute, mean, L (p = 0.006)	2.39	2.82
DLCO absolute, mean, mmHg/mL/min (p = 0.50)	13.36	13.36
FVC percent predicted, mean, % (p = 0.012)	67.2%	75.0%
DLCO percent predicted, mean, % (p = 0.35)	56.8%	55.4%
Diagnosed by lung biopsy alone, n (%)	4 (10%)	2 (4%)
Diagnosed by HRCT alone, n (%)	6 (15%)	5 (10%)
Pulmonary hypertension, n (%)	2 (5%)	8 (16%)
Coexisting emphysema, n (%)	1 (3%)	4 (8%)
GAP stage 1 (3.1% mortality at 1 year for FHP, 5.6% mortality at 1 year for IPF)	21	27
GAP stage 2 (8.8 % mortality at 1 year for FHP, 16.2% mortality at 1 year for IPF)	14	19
GAP stage 3 (18.2% mortality at 1 year for FHP, 39.2% mortality at 1 year for IPF)	4	4

**Table 2:** GAP-IPF scores for FHP and IPF cohorts* *Demonstrating similarities in disease severity based on an externally validated study utilizing objective measures including gender, age, predicted FVC, and predicted DLCO [13] DLCO: diffusion capacity of lungs for carbon monoxide; FHP: fibrotic hypersensitivity pneumonitis; FVC: forced vital capacity; GAP: gender, age, and physiology; FHP: fibrotic hypersensitivity pneumonitis; IPF: idiopathic pulmonary fibrosis

	GAP stage 1 (score 1 or less, 5.6% mortality at 1 year)	GAP stage 1 (score 2-3, 5.6% mortality at 1 year)	GAP stage 2 (score 4-5, 16.2% mortality at 1 year)	GAP stage 3 (score 5 or more, 39.2% mortality at 1 year)
FHP	2 (5%)	19 (49%)	14 (36%)	4 (10%)
IPF	1 (2%)	26 (52%)	19 (38%)	4 (8%)

**Table 3:** ILD-GAP scores for FHP and IPF cohorts* *Also externally validated but has adjustments in criteria for FHP (-2 points for FHP in this model) to reflect lesser disease mortality [14] FHP: fibrotic hypersensitivity pneumonitis; GAP: gender, age, and physiology; ILD: interstitial lung disease; IPF: idiopathic pulmonary fibrosis

	ILD-GAP stage 1 (score 1 or less, 3.1% mortality at 1 year)	ILD-GAP stage 2 (score 2-3, 8.8% mortality at 1 year)	GAP stage 3 (score 4-5, 18.2% mortality at 1 year)	GAP stage 4 (score 5 or more, 33.5% mortality at 1 year)
FHP	21 (54%)	14 (36%)	4 (10%)	0
IPF	1 (2%)	26 (52%)	19 (38%)	4 (8%)

There was a median of 32 days and a mean of 36 days between PFTs and diagnosis dates; 89 patients met the inclusion criteria: 39 (44%) with FHP and 50 (56%) with IPF. The mean baseline F/D (%pred) ratio was significantly lower for patients with FHP (M = 1.24, 95% CI: 1.14, 1.33) than for patients with IPF (M = 1.44, 95% CI: 1.31, 1.57, T(87) = 2.23, p = 0.014). The mean baseline F/D (absolute) ratio was also significantly lower for patients with FHP (M (absolute) = 0.187, SD = 0.050, 95% CI: 0.171, 0.203) than for patients with IPF (M (absolute) = 0.223, SD = 0.064, 95% CI: 0.205, 0.241, T(87) = 2.90, p = 0.002).

**Figure 1:**
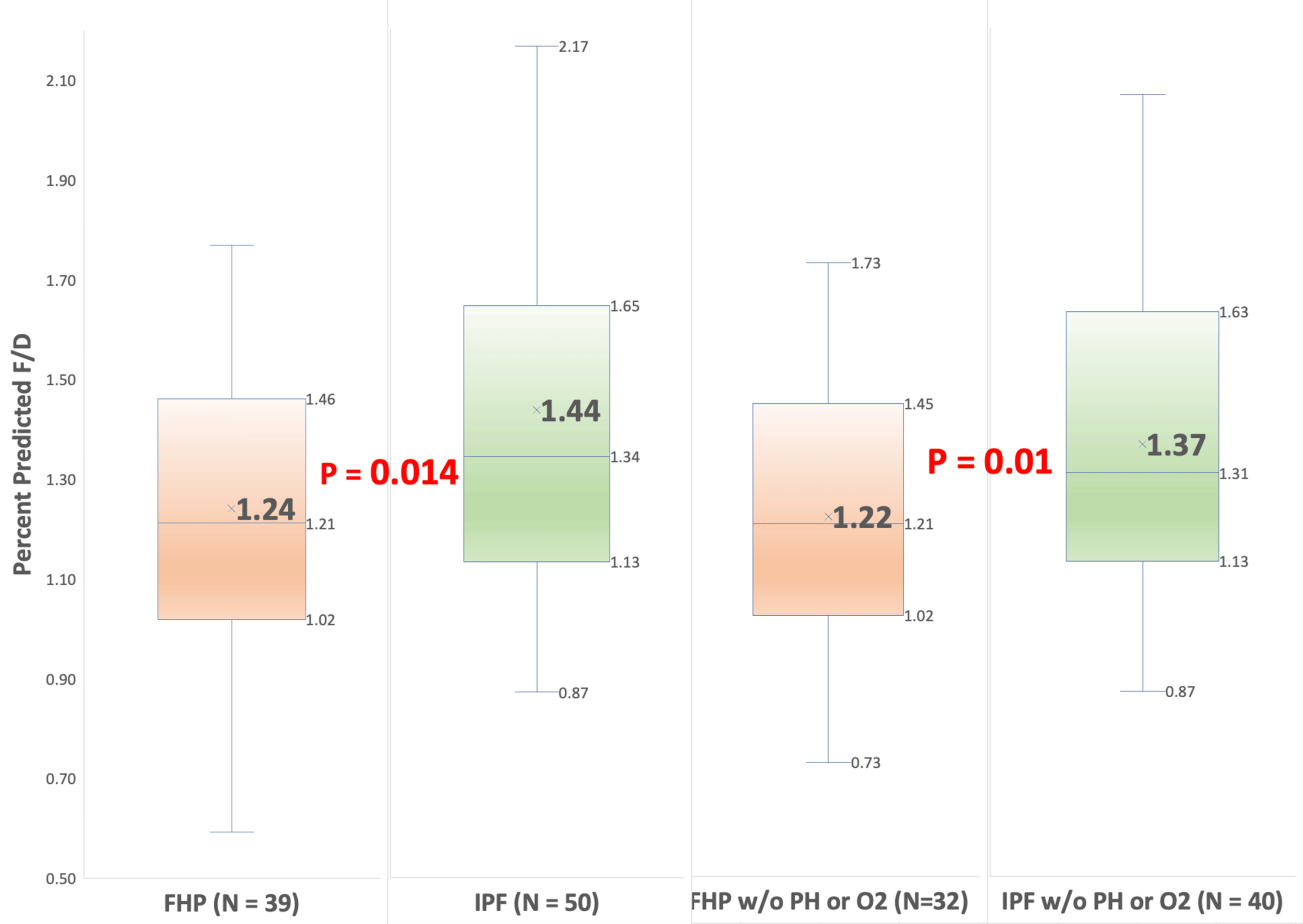
Box-and-whisker plot with F/D ratio calculated using percent predicted values for FVC and DLCO Primary analysis: FHP (M = 1.24, SD = 0.30, IQR = Q3(1.46) - Q1(1.02)), IPF (M = 1.44, SD = 0.47, IQR = Q3(1.65) - Q1(1.13), T(87) = 2.23, p = 0.014). Secondary analysis: FHP (M = 1.22, SD = 0.29, IQR = Q3(1.45) - Q1(0.97)), IPF (M = 1.37, SD = 0.32, IQR = Q3(1.63) - Q1(1.14)), T (70) = 2.37, p = 0.01) DLCO: diffusion capacity of lungs for carbon monoxide; FHP: fibrotic hypersensitivity pneumonitis; FVC: forced vital capacity; IPF: idiopathic pulmonary fibrosis; IQR: interquartile range; SD: standard deviation

**Figure 2:**
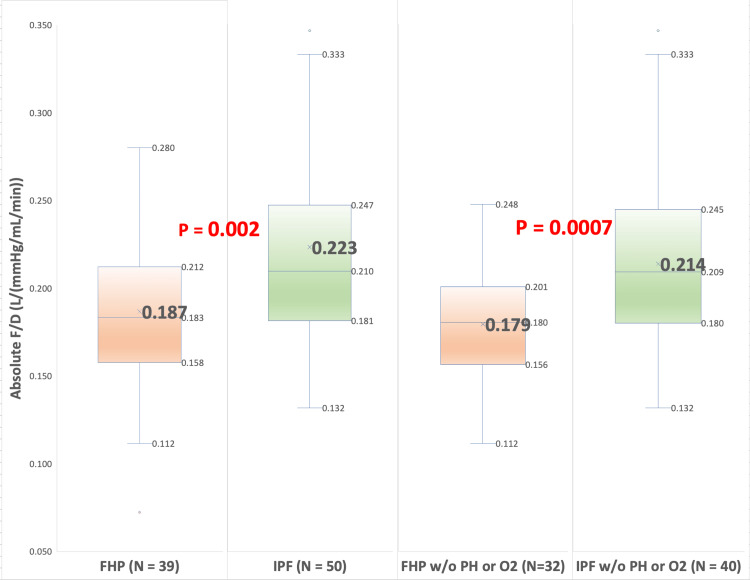
Box-and-whisker plot with F/D ratio calculated using absolute values for FVC and DLCO Primary analysis: FHP (M (absolute) = 0.187, SD = 0.050, IQR = Q3(0.158) - Q1(0.212)), IPF (M (absolute) = 0.223, SD = 0.064, IQR = Q3(0.181) - Q1(0.247), T(87) = 2.90, p = 0.002). Secondary analysis: FHP (M (absolute) = 0.179, SD = 0.033, IQR = Q3(0.156)-Q1(0.201)), IPF M (absolute) = 0.214, SD = 0.050, IQR = Q3(0.180) - Q1(0.245), T(70) = 3.32, p = 0.0007) DLCO: diffusion capacity of lungs for carbon monoxide; FHP: fibrotic hypersensitivity pneumonitis; FVC: forced vital capacity; IPF: idiopathic pulmonary fibrosis; IQR: interquartile range; SD: standard deviation

Secondary analysis was performed by excluding patients with coexisting pulmonary hypertension and resting hypoxemia at the time of PFTs, which would decrease and increase DLCO, respectively. This analysis yielded 72 patients: 32 (44%) with FHP and 40 (56%) with IPF. The mean baseline F/D (%pred) ratio was significantly lower for patients with FHP (M = 1.22, 95% CI: 1.12, 1.31) than for patients with IPF (M = 1.37, 95% CI: 1.27, 1.46, T (70) = 2.37, p = 0.01). The mean baseline F/D (absolute) ratio was also significantly lower for patients with FHP (M (absolute) = 0.179, SD = 0.033, 95% CI: 0.168, 0.191) than for patients with IPF (M (absolute) = 0.214, SD = 0.050, 95% CI: 0.198, 0.229, T(70) = 3.32, p = 0.0007). Figure 1 represents results in a box-and-whisker plot with F/D calculated by using the percentage of predicted values for FVC and DLCO. Figure 2 also shows a box-and-whisker plot with F/D calculated using absolute values for FVC and DLCO.

## Discussion

In this study, the baseline F/D ratios for patients with probable to definite FHP and IPF were examined. In the primary analysis, the F/D ratio was significantly lower in patients with FHP compared to those with IPF. After excluding potential confounders of pulmonary hypertension and supplemental oxygen use at rest, F/D ratios were still significantly lower in patients with FHP than those with IPF. A lower baseline F/D ratio therefore shows the potential to increase clinical suspicion for FHP over IPF. While previous studies have examined F/D ratios in predicting pulmonary hypertension in ILDs, chronic obstructive pulmonary disease, and systemic sclerosis, no studies are known to have directly evaluated the F/D ratio in patients with confirmed diagnoses of FHP or IPF [15-17]. Furthermore, no studies are known to have compared F/D ratios between FHP and IPF patients or demonstrate that FHP patients have lower baseline F/D ratios than IPF patients.

Increased suspicion for one disease over the other and differentiating FHP from IPF and vice versa are key in terms of pathophysiology, treatment, and prognoses. FHP is caused by a dysregulated immune response and fibrosis linked to exposures to environmental antigens from microorganisms, fungi, or animals among many others, while IPF is a progressive idiopathic fibroproliferative disease of the lungs [1-3]. Both diseases involve fibrosis of the lung parenchyma and lead to restrictive patterns on PFTs with reduced total lung capacity, FVC, and DLCO. FHP may respond more favorably to judicious anti-inflammatory or immunosuppressive therapies and have a better prognosis than IPF [18]. In contrast, these treatments have been proven to cause considerable harm in patients with IPF, who should be treated with antifibrotics and have a poor prognosis of three to five years after diagnosis with treatment [19]. Importantly, the fibrosis and disease in FHP is bronchiolocentric, or focused around the bronchioles or small airways, compared to IPF, where the fibrosis is more global and diffuse. This may explain the lower F/D ratios in patients with FHP than those with IPF as DLCO could be more negatively affected in patients with IPF due to more diffuse involvement of the lung parenchyma. 

While these diseases have distinct etiologies, treatments, and prognoses, FHP and IPF remain difficult to differentiate due to overlapping symptoms, physiology, radiology, and histopathology. Patients with either disease typically present with progressive dyspnea, cough, and declining lung function. IPF is classically associated with a UIP, or usual interstitial pneumonia, pattern, on HRCT and histopathology, but these patterns can sometimes be seen in FHP as well [1-3]. Histopathology via lung biopsy is ideal for diagnosis but may also be prohibitive due to advanced age, comorbidities, and disease severity. A timely, accurate, and less invasive way of differentiating FHP from IPF is essential to avoid misdiagnosis and mismanagement and improve patient outcomes. Pulmonary function testing is routinely performed on patients with lung diseases, and in the case of FHP and IPF, regularly performed to establish baseline lung function and monitor the progression of diseases and treatment through trends of FVC, DLCO, and more [1-3]. Therefore, a lower baseline F/D ratio may be a new, readily available index that fills this role by potentially increasing clinical suspicion of FHP versus IPF through the use of routine, non-invasive testing via PFTs.

The F/D ratios were calculated using both the percentage of predicted and absolute values for FVC and DLCO. Initially, the F/D ratios were calculated with the FVC and DLCO percentage of predicted values as one of the aims of the study was to ensure this ratio could be practically calculated in clinical practice and thus used the larger numbers associated with such values. As mentioned, there is evidence against the use of race and ethnicity in PFTs as supported by the ATS statement. For example, studies have shown that mortality becomes more similar between Black and White individuals if a single reference equation rather than traditional race-specific equations are used [12]. Additionally, another study showed that the prevalence of emphysema in Black and White individuals was better matched based on predicted forced expiratory volume in one second using a race-neutral equation [20].

Notably, the patient population examined in this study did not include Black individuals. However, there were 10 out of 39 patients with FHP and seven out of 50 patients with IPF with unknown race, which could potentially consist of Black persons, but this data is limited by the available information in the electronic medical record. Hence, the F/D ratio calculated using the percentage predicted values for FVC and DLCO was likely less influenced by the consideration of race in the traditional race-specific equations. Furthermore, using the absolute values for FVC and DLCO resulted in a more statistically significant difference (p = 0.0007) when comparing the mean F/D ratios in patients with FHP and patients with IPF, and the F/D ratio was still lower.

This study does have certain limitations. As a retrospective chart review performed at a single center, smaller sample size and correlation of PFTs and therefore FVC and DLCO values with disease onset and diagnosis are the main drawbacks. Although the results were statistically significant, a larger sample size could allow for more generalizability of the results and clinical use of the F/D ratio. While the PFTs used for data collection and analysis were within six months of a confirmed diagnosis of either FHP or IPF, this does not necessarily correlate with the onset of the disease and symptoms for every patient. For example, a patient may present to the clinic several months or years after symptoms develop and have their first PFTs done, and be diagnosed relatively soon after. However, their F/D ratio at this time may not be the same as when their disease first truly began. As diseases for both FHP and IPF become more severe, it is expected that F/D ratios will be higher for both.

The above-mentioned limitation of the study can be reflected in the data, where the average baseline FVC absolute and percentage predicted values were statistically lower in the FHP cohort compared to the IPF cohort (FHP average FVC Abs = 2.39, IPF average FVC Abs = 2.82, p = 0.006; FHP average FVC %pp = 67%, IPF average FVC %pp = 75% p = 0.01). In relation to the similar average DLCO values for both groups (FHP average DLCO Abs = 13.36, IPF average DLCO Abs = 13.36, p =0.50; FHP average DLCO %pp = 56.8, IPF average DLCO %pp = 55.4, p = 0.35), it is uncertain whether FHP patients in this cohort had a lower baseline FVC value due to the disease process itself or if the baseline disease was more severe. However, stratification through the externally validated GAP-IPF score elucidates that disease severity between the FHP and IPF cohorts may be more alike as the percentages in each scoring stage were similar and the GAP-IPF index considers objective lung function data of FVC and DLCO in addition to gender and age [13]. The ILD-GAP index was created to estimate mortality in other ILDs using similar criteria as GAP-IPF but with adjustments in observed mortality seen in other cohorts, such as FHP [14]. With ILD-GAP, there are differences in mortality when comparing FHP and IPF scores as intended, with the majority of the FHP cohort in stage 1 with 3.1% one-year mortality versus in IPF, where the majority were classified as stage 2 with 8.8% one-year mortality.

There was also a lack of standard evaluation for pulmonary hypertension in all patients in these cohorts; 37 patients out of 90 total (22 FHP and 15 IPF patients) did not have an echocardiogram or right heart catheterization performed, which are two standard diagnostic tests that can evaluate for pulmonary hypertension. Thus, some patients may or may not have been excluded in the secondary analysis where patients with pulmonary hypertension were excluded.

## Conclusions

In patients with probable to definite FHP versus IPF, the baseline FVC/DLCO, or F/D ratios, were significantly lower in those with FHP than IPF, even after excluding patients with coexisting pulmonary hypertension and resting hypoxemia. A lower baseline F/D ratio therefore may be a novel, clinic-ready index to heighten clinical suspicion for FHP compared to IPF as these diseases can be difficult to differentiate even with an extensive workup due to overlapping presentations, pathophysiology, and imaging and histopathology findings. The F/D ratio can also potentially obviate the need for invasive diagnostics such as lung biopsies and reduce the time and procedural burden on patients. Future larger prospective studies involving multiple centers are needed to validate these findings and to develop cut-off values for true clinical use in differentiating FHP and IPF. Additionally, future studies should involve a detailed analysis of HRCT and biopsy findings to find correlates to explain the pathophysiology of lower F/D ratios in patients with FHP.
